# Clinical impact of diabetes mellitus in patients undergoing transcatheter aortic valve replacement

**DOI:** 10.1186/s12933-015-0291-3

**Published:** 2015-10-01

**Authors:** Anat Berkovitch, Amit Segev, Israel Barbash, Yoni Grossman, Elad Maor, Aharon Erez, Ehud Regev, Noam Fink, Israel Mazin, Ashraf Hamdan, Ilan Goldenberg, Ilan Hay, Dan Spiegelstien, Victor Guetta, Paul Fefer

**Affiliations:** Leviev Heart Center, Chaim Sheba Medical Center, 52621 Tel Hashomer, Israel; Department of Internal Medicine D, Chaim Sheba Medical Center, Tel Hashomer, Israel; Pinchas Borenstein Talpiot Medical Leadership Program, Chaim Sheba Medical Center, Tel Hashomer, Israel; Sackler School of Medicine, Tel-Aviv University, Tel Aviv, Israel

**Keywords:** Diabetes mellitus, Trans-arterial valve replacement, Prognosis, Valvular disease

## Abstract

**Background:**

Diabetes mellitus (DM) and aortic stenosis (AS) are frequent findings in the elderly population. Data regarding the influence of DM on the outcomes of patients undergoing transcatheter aortic valve replacement (TAVR) due to AS are limited. The aim of this study was to examine the impact of DM on TAVR outcomes.

**Methods:**

We investigated 443 patients with severe AS undergoing TAVR. Subjects were divided into insulin-dependent diabetic mellitus (IDDM) patients (N = 44), non-dependent insulin diabetic mellitus (NIDDM) patients (N = 114) and non-diabetics (N = 285) of whom 31 (74 %), 86 (79 %) and 209 (76 %) respectively had trans-femoral TAVR. Peri-procedural complications and outcomes were recorded according to the Valve Academic Research Consortium-2 criteria.

**Results:**

Patients with IDDM as well as NIDDM demonstrated similar complication rates compared with non-diabetic patients, except for acute kidney injury (AKI) grade 3 [4 (2 %) and 3 (3 %) vs. 1 (0.4 %) respectively, p = 0.032]. Kaplan–Meier survival analysis showed that DM, regardless of the type of treatment, was not associated with increased 2 years mortality (Log-rank p value 0.44). Multivariate cox regression analysis adjusted for age, gender, coronary artery disease, DM, AKI3, hypertension, chronic renal failure and peripheral vascular disease found that AKI3 was associated with increased risk of 2 years mortality [HR = 7.35, 95 % CI 2.16–25.07, p = 0.001] whereas female gender was found as a protective factor [HR = 0.47, 95 % CI 0.28–0.8, p = 0.005], and DM was not associated with increased risk.

**Conclusions:**

Following TAVR, DM patients seem to have similar peri-procedural and mid-term outcomes compared with patients without DM, while IDDM patients seem to suffer greater incidence of AKI. Further research in larger cohorts of patients is needed to validate our results.

**Electronic supplementary material:**

The online version of this article (doi:10.1186/s12933-015-0291-3) contains supplementary material, which is available to authorized users.

## Background

Trans-catheter aortic valve replacement (TAVR), previously reserved for surgically high-risk patients with symptomatic aortic stenosis (AS), is now being utilized in a wider range of patients [1, 2]. Patients treated with TAVR have shown significant alleviation of symptoms and improved functional status [3, 4].

Diabetes mellitus (DM) and AS are both prevalent in the elderly population, and symptomatic AS has been found to be associated with DM [5]. Following cardiac surgery, patients with DM have been shown to suffer higher rates of renal dysfunction, need for blood transfusions and lung complications. Furthermore, DM patients undergoing TAVR have been shown to have less complications than DM patients undergoing surgical aortic valve replacement (SAVR) [6], and while mortality rates in patients undergoing TAVR have been shown to be equivalent [7] or reduced [8] compared with patients treated with SAVR, little is known about the impact of DM on the clinical outcomes of patients undergoing TAVR compared with the non-DM population. The aim of the current study was to investigate the clinical outcomes and complication rates among DM patients undergoing TAVR.

## Methods

The study included 443 consecutive patients undergoing TAVR at the Sheba Medical Center between January 2008 and December 2014, of which 158 (36 %) had DM. We analyzed separately DM patients treated with insulin (IDDM) (44 [28 %]) and those treated either by diet or oral anti-diabetic agents (114 [72 %]). Candidates for TAVI were evaluated separately by an interventional cardiologist and by a cardiac surgeon. Cases were then discussed by the heart team which convenes on a weekly basis. Final decision to advise TAVR vs. SAVR was made by the heart team based on suitability for TAVI and surgical risk.

Baseline clinical data and chronic medication use were obtained from patients’ electronic records. Patients with impaired renal function (creatinine >1.2 mg/dl) received peri-procedural balanced intravenous volume expansion with isotonic sodium chloride [9]. Peri-procedural complications were prospectively recorded according to the Valve Academic Research Consortium (VARC)-2 criteria. Mortality rates were ascertained with the Israeli Ministry of Interior mortality database through January 2015.

The study was approved by the local institutional review board (clinicaltrial.gov: SHEBA-13-0685-IB-CTIL).

### Definitions and outcome measures

Patients were considered to have DM if they fulfilled at least one of the followings: (1) HbA1c >6.5 % at admission, (2) a random plasma glucose level higher than 200 mg/dl in the presence of symptoms, (3) use of glucose lowering medications. All DM patients had type 2 DM. Renal dysfunction was defined as a serum creatinine level greater than 1.4 mg %.

The primary outcome of the current study was all cause mortality during a follow-up period of 2 years. Median follow-up time for the entire study population was 599 days. Secondary outcomes were defined as any VARC-2 defined peri-procedural complication.

### Statistical analysis

Continuous data were compared with Student’s t-test and one-way ANOVA. Categorical data were compared with the use of Pearson Chi square test.

Kaplan–Meier survival analysis was used to descriptively show the association between DM and subsequent mortality. Multivariate cox regression adjusted for age, gender, coronary artery disease, DM, AKI3, hypertension, chronic renal failure and peripheral vascular disease was performed in order to find risk factors for mortality. Statistical significance was accepted for a two-sided p < 0.05. The statistical analyses were performed with IBM SPSS version 20.0 (Chicago, IL, USA).

## Results

Of 443 patients included in the study 158 (36 %) had DM. These patients were compared with the 285 (64 %) patients without DM. Of the 158 diabetic patients 44 (28 %) were treated with insulin, while the others were treated either orally or by diet. Baseline clinical and laboratory characteristic by DM status are presented in Table 1. Notably, DM subjects were on average 2 years younger than non-diabetic patients and had a greater incidence of dyslipidemia. Subjects with IDDM had significantly greater incidence of baseline renal dysfunction. However, no other major differences in baseline characteristics were noted between groups, including STS score, EUROSCORE, NYHA class, or presence of coronary artery disease.

**Table 1:** Baseline characteristics

	Non-diabetic (N = 285)	Diabetic (N = 158)		P-value
		NIDDM (N = 114)	IDDM (N = 44)	
Age	81.8 ± 7.5	79.8 ± 7.5	79.4 ± 7.7	0.016
Male	128 (46)	53 (46)	23 (52)	0.71
Hemoglobin	11.5 (1.4)	11.6 (1.4)	12.6 (2.2)	0.18
Glucose	91 (13)	97 (21)	95 (34)	0.21
Renal dysfunction	46 (17)	21 (20)	23 (59)	<0.001
Hypertension	232 (84)	103 (94)	34 (83)	0.032
Dyslipidemia	199 (72)	90 (83)	34 (85)	0.036
Coronary artery disease	124 (46)	48 (45)	24 (60)	0.22
CHF-NYHA class III–IV	165 (58)	62 (54)	23 (52)	0.64
Atrial fibrillation/flutter	66 (27)	29 (28)	13 (33)	0.71
Peripheral vascular disease	33 (12)	13 (12)	6 (15)	0.85
Current smoker	11 (4)	2 (2)	2 (5)	0.53
COPD	41 (15)	19 (18)	11 (28)	0.13
s/p CABG	57 (21)	22 (21)	10 (26)	0.8
s/p PCI	73 (27)	30 (29)	14 (36)	0.55
Pacemaker	21 (8)	7 (7)	2 (5)	0.79
STS mortality	5.3 ± 3.7	5.2 ± 2.9	7.6 ± 5.6	0.21
EUROSCORE 2	4.9 ± 5.1	4.7 ± 5.3	9.1 ± 8.7	0.16
HbA1c	5.7 ± 0.43	6.8 ± 1.00	7.9 ± 1.47	<0.001

All values are expressed as mean ± SD or N (%)

*NIDDM* non-insulin dependent diabetes mellitus, *IDDM* insulin dependent diabetes mellitus, *CHF* congestive heart failure, *NYHA* New York Heart Association, *COPD* chronic obstructive lung disease, *CABG* coronary artery bypass surgery, *PCI* percutaneous coronary intervention, *STS* Society of Thoracic Surgeons

Procedural characteristics are summarized in Table 2. In most patients TAVR was performed via the trans-femoral approach (76 %) using conscious sedation (63 %). There were no differences between groups regarding the vascular approach, type of anesthesia, valve type or valve size used.

**Table 2:** Procedural characteristics of TAVR patients

	Non-diabetic (N = 285)	Diabetic (N = 158)		P-value
		NIDDM (N = 114)	IDDM (N = 44)	
Approach				
Trans-femoral	209 (76)	86 (79)	31 (74)	0.53
Trans-apical	49 (18)	19 (17)	9 (21)	
Other	17 (6)	3 (3)	2 (5)	
Anesthesia				
General	89 (39)	34 (34)	12 (36)	0.55
Conscious sedation	140 (61)	66 (66)	21 (61)	
Valve type				
SAPIEN XT	109 (41)	48 (45)	18 (45)	0.35
CoreValve	159 (58)	55 (51)	20 (50)	
Other	5 (1)	2 (2)	0 (0)	
Valve size				
23 mm	52 (20)	19 (18)	9 (24)	0.77
25 mm	0 (0)	1 (1)	0 (0)	
26 mm	106 (41)	45 (44)	13 (35)	
29 mm	93 (36)	34 (33)	13 (35)	
31 mm	6 (2)	4 (4)	2 (5)	
Valve-in-valve	25 (9)	12 (11)	8 (20)	0.11
Procedural success	262 (98)	103 (97)	38 (100)	0.59
Pre procedural EF	55 ± 12	56 ± 12	54 ± 12	0.48
Pre-procedural moderate to severe MR	74 (26)	26 (23)	10 (23)	0.76
Post-procedural moderate to severe AR	37 (13)	14 (12)	2 (5)	0.27

All values expressed as N (%)

*NIDDM* non-insulin dependent diabetes mellitus, *IDDM* insulin dependent diabetes mellitus

**Fig. 1:**
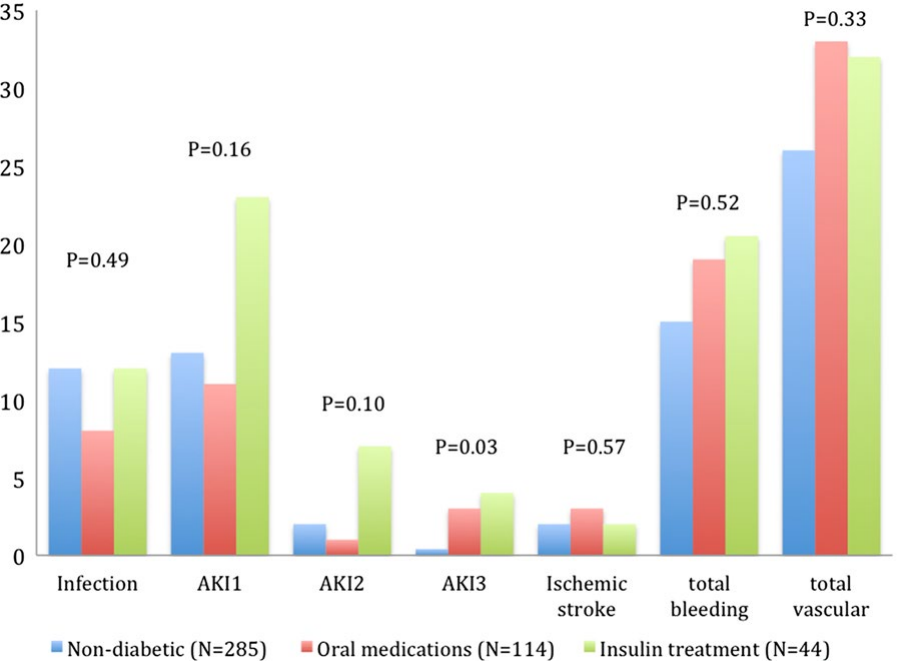
Systemic complication rates. The figure shows the systemic complication rates (in percentage) according to diabetic status

Diabetic patients demonstrated similar complication rates compared with non-diabetic patients (Table 3, Fig. 1), except for acute kidney injury (AKI) grade 3, which was more common in the IDDM group [1 (0.4 %) vs. 3 (3 %) and 2 (4 %), for non-diabetic, NIDDM, and IDDM respectively, p = 0.032]. No other significant differences were noted between groups.

**Table 3:** Cardiac complications

Complication	Non-diabetic (N = 285)	Diabetic (N = 158)		P-value
		NIDDM (N = 114)	IDDM (N = 44)	
30-days mortality	12 (4.2)	3 (2.6)	2 (4.5)	0.73
6-months mortality	28 (9.8)	5 (4.4)	3 (6.8)	0.18
1-year mortality	39 (13.7)	11 (9.6)	4 (9.1)	0.43
Conversion to open surgery	5 (2)	0 (0)	0 (0)	0.24
Unplanned cardiopulmonary bypass	3 (1)	0 (0)	0 (0)	0.43
Coronary obstruction	1 (0.4)	1 (0.9)	0 (0)	0.7
Cardiac tamponade	8 (3)	4 (4)	1(2)	0.91
Valve malpositioning	11 (4)	7 (6)	1 (2)	0.5
Valve migration	11 (4)	5 (4)	1 (2)	0.83
Peri-procedural MI (≤72 h post procedure)	9 (3)	1(1)	1 (2)	0.41
Spontaneous MI (≤72 h post procedure)	3 (1)	0 (0)	1 (2)	0.36
Transient atrio-ventricular block	21 (9)	9 (9)	3 (7)	0.96
Permanent atrio-ventricular block	41 (17)	18 (18)	5 (12)	0.72
Permanent pacemaker implantation	55 (22)	24 (23)	8 (19)	0.86
New LBBB	78 (32)	30 (29)	8 (19)	0.27
New RBBB	3 (1)	1 (1)	0 (0)	0.77
New onset AF	27 (11)	17 (16)	3 (7)	0.22
AF episode in patient with history of AF	13 (5)	7 (7)	3 (7)	0.78
Heart failure post-procedure	23 (9)	12 (12)	7 (17)	0.33

All values expressed as N (%)

*NIDDM* non-insulin dependent diabetes mellitus, *IDDM* insulin dependent diabetes mellitus, *MI* myocardial infarction, *LBBB* left bundle branch block, *RBBB* right bundle branch block, *AF* atrial fibrillation

**Fig. 2:**
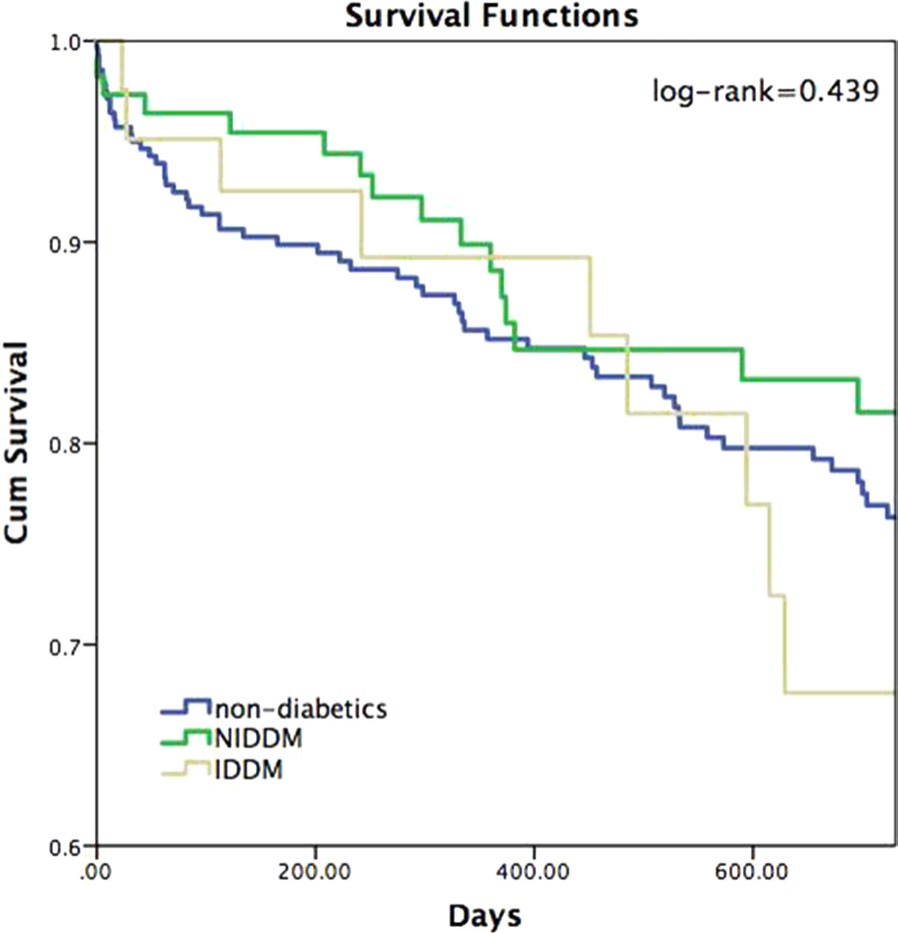
Kaplan–Meier analysis for 2 years survival. The Kaplan–Meier analysis was used to compare the probability of 2 years survival according to diabetic status. P value (Log rank) = 0.439

The primary study outcome occurred in 108 (24.4 %) subjects. Kaplan–Meier survival analysis (Fig. 2) showed that the cumulative probability for mortality through 2 years of follow-up was not different between the groups (p-value Log rank = 0.439), although there was a trend towards higher mortality among patients with IDDM.

Multivariate cox regression adjusted for age, gender, coronary artery disease, DM, AKI3, hypertension, chronic renal failure and peripheral vascular disease found that AKI3 [HR = 7.35, 95 % CI 2.16–25.07, p = 0.001] and peripheral vascular disease [HR = 1.95, 95 % CI 1.12–3.39, p = 0.019] were associated with increased risk of 2-year mortality. Female gender was found to be a protective factor [HR = 0.47, 95 % CI 0.28–0.8, p = 0.005] and DM was not associated with increased risk (Table 4).

**Table 4:** Multivariate cox regression analysis for 2-year mortality

Factor	HR	95 % confidence interval	P value
Acute kidney injury 3	7.35	2.15–25.07	0.001
Peripheral vascular disease	1.95	1.12–3.39	0.019
Chronic renal failure	1.59	0.96–2.62	0.07
Diabetes mellitus	0.83	0.49–1.40	0.48
Female gender	0.47	0.28–0.80	0.005

Analysis was further adjusted for age, coronary artery disease and hypertension

We performed two additional sub-group analyses: first we analyzed outcomes of diabetic patients based on their HbA1C levels. Documented HbA1C levels during hospitalization were available in 105 patients with DM. Outcomes of well-controlled diabetic patients with HbA1C levels below 7 % were equivalent to diabetic patients with HbA1C levels equal to or above 7 % (Additional file 1: Table S1). While numerically higher 1 year mortality rates were noted among DM patients with HbA1c levels above 7 % compared with those below 7 % [2 (4 %) vs 5 (9 %), p = 0.23], this finding did not reach statistical significance, probably due to the relatively small group sample.

## Discussion

The major finding of our study is that DM patients undergoing TAVR seem to have favorable outcomes with similar short and mid-term mortality rates compared with non-DM patients. Patients with IDDM in our study had greater incidence of renal insufficiency at baseline and both DM groups suffered from increased rate of AKI after the procedure, however other peri-procedural complications occurred equally among DM and non-DM patients.

Up to 40 % of older individuals have DM [10]. Elderly DM patients have unique characteristics and suffer from higher cardiovascular disease rates compared to younger DM patients [11]. However, limited data exists with respect to the effect of DM on mortality after TAVR. Our findings are supported by two previous studies: Minha et al. [12] followed 499 patients who had TAVR and concluded that DM was not associated with poor outcome. However in this study patients were not divided according to treatment type (insulin vs. oral hypoglycemic agents). Conrotto et al. [13] examined 511 patients undergoing TAVR, of whom 150 had DM, and also found no difference regarding the short and mid-term effect of DM on mortality rates in these patients. A number of studies suggest differential outcomes in DM patients: Tamburino et al. [14], in a study of 663 patients after TAVR found a higher incidence of DM among those who died and argued that DM at baseline may be associated with increased mortality. However this study failed to compare baseline characteristics between DM and non-DM subjects and is inherently biased in that it examined characteristics of patients who died following the procedure. Similarly, Puls et al. [15] reported poor outcome among 108 DM patients. However, in contrast to our series, most TAVR were performed by the transapical approach and their total mortality rates were much higher than those found in our cohort, suggesting that these patients had greater baseline risk.

While we failed to demonstrate statistically significant differences in mortality rates between IDDM and NIDDM patients, Kaplan–Meier analysis did suggest a trend towards worse outcomes among IDDM subjects. However, our sample size is inadequate to properly address mortality between groups.

Acute kidney injury rates were significantly higher among DM patients, especially the IDDM group. This finding is not surprising given the higher CRF rates at baseline. Previous studies have found CRF and AKI injury to be predictors for in-hospital mortality, 30 days mortality and 1 year mortality post-TAVR [16–19]. Our findings are similar to those mentioned above. However, and despite having higher rates of AKI, IDDM patients in our study did not demonstrate significantly higher mortality rates when compared with NIDDM patients or with non-diabetic patients. A possible explanation for this discrepancy may be the small number of IDDM or the relatively small number of events (AKI) in the population which failed to reach statistical significance.

### Limitations

This paper is based on a moderately sized cohort emanating from a single center. The limited number of enrolled patients does not allow assessment of specific outcome measures and sub-group analysis and avoids the potential for a propensity-matched evaluation or use of post hoc comparisons between the three groups due to high-risk for type II errors. Larger cohorts are necessary to further assess the prognostic impact of diabetes mellitus on outcome after TAVR.

## Conclusions

Diabetic patients seem to have favorable short- and mid-term outcomes following TAVR which are similar to patients without DM. Insulin treated DM patients may have greater incidence of AKI, and appropriate peri-procedural strategies should be employed to minimize this risk in these patients.

## Additional file

10.1186/s12933-015-0291-3 Complication rates based on HbA1c levels.

## Abbreviations

DM: diabetes mellitus
TAVR: trans-catheter aortic valve replacement
AKI: acute kidney injury
VARC: Valve Academic Research Consortium
NYHA: New York Heart Association

## References

[1] Gotzmann M, Pljakic A, Bojara W, Lindstaedt M, Ewers A, Germing A, Mügge A (2011). Transcatheter aortic valve implantation in patients with severe symptomatic aortic valve stenosis-predictors of mortality and poor treatment response. Am Heart J.

[2] Poliacikova P, Cockburn J, Pareek N, James R, Lee L, Trivedi U, De Belder A, Hildick-smith D (2013). Prognostic impact of pre-existing right ventricular dysfunction on the outcome of transcatheter aortic valve implantation. J Invasive Cardiol.

[3] Webb JG, Pasupati S, Humphries K, Thompson C, Altwegg L, Moss R, Sinhal A, Carere RG, Munt B, Ricci D, Ye J, Cheung A, Lichtenstein SV (2007). Percutaneous transarterial aortic valve replacement in selected high-risk patients with aortic stenosis. Circulation.

[4] Grube E, Schuler G, Buellesfeld L, Gerckens U, Linke A, Wenaweser P, Sauren B, Mohr FW, Walther T, Zickmann B, Iversen S, Felderhoff T, Cartier R, Bonan R (2007). Percutaneous aortic valve replacement for severe aortic stenosis in high-risk patients using the second- and current third-generation self-expanding CoreValve prosthesis. Device success and 30-day clinical outcome. J Am Coll Cardiol.

[5] Falcão-Pires I, Hamdani N, Borbély A, Gavina C, Schalkwijk CG, van der Velden J, van Heerebeek L, Stienen GJM, Niessen HWM, Leite-Moreira AF, Paulus WJ (2011). Diabetes mellitus worsens diastolic left ventricular dysfunction in aortic stenosis through altered myocardial structure and cardiomyocyte stiffness. Circulation.

[6] Lindman BR, Pibarot P, Arnold SV, Suri R, McAndrew TC, Maniar HS, Zajarias A, Kodali S, Kirtane AJ, Thourani VH, Tuzcu EM, Svensson LG, Waksman R, Smith CR, Leon MB (2013). Transcatheter versus surgical aortic valve replacement in patients with diabetes and severe aortic stenosis at high risk for surgery: an analysis of the PARTNER trial. J Am Coll Cardiol.

[7] Smith CR, Leon MB, Mack MJ, Miller DC, Moses JW, Svensson LG, Tuzcu EM, Webb JG, Fontana GP, Makkar RR, Williams M, Dewey T, Kapadia S, Babaliaros V, Thourani VH, Corso P, Pichard AD, Bavaria JE, Herrmann HC, Akin JJ, Anderson WN, Wang D, Pocock SJ (2011). Transcatheter versus surgical aortic-valve replacement in high-risk patients. N Engl J Med.

[8] Reardon M, et al. A randomized comparison of self-expanding transcatheter and surgical aortic valve replacement in patients with severe aortic stenosis deemed at increased risk for surgery 2-year outcomes. In: Presented at ACC, San Diego, CA. 2015. 10.1016/j.jacc.2015.07.042 26383718

[9] Fliser D, Laville M, Covic A, Fouque D, Vanholder R, Juillard L, Van Biesen W (2012). A European Renal Best Practice (ERBP) position statement on the kidney disease improving global outcomes (KDIGO) clinical practice guidelines on acute kidney injury: part 1: definitions, conservative management and contrast-induced nephropathy. Nephrol Dial Transpl.

[10] Sinclair A, Morley JE, Rodriguez-Mañas L, Paolisso G, Bayer T, Zeyfang A, Bourdel-Marchasson I, Vischer U, Woo J, Chapman I, Dunning T, Meneilly G, Rodriguez-Saldana J, Gutierrez Robledo LM, Cukierman-Yaffe T, Gadsby R, Schernthaner G, Lorig K (2012). Diabetes mellitus in older people: position statement on behalf of the International Association of Gerontology and Geriatrics (IAGG), the European Diabetes Working Party for Older People (EDWPOP), and the International Task Force of Experts in Diabetes. J Am Med Dir Assoc.

[11] Bethel MA, Sloan FA, Belsky D, Feinglos MN (2007). Longitudinal incidence and prevalence of adverse outcomes of diabetes mellitus in elderly patients. Arch Intern Med.

[12] Minha S, Magalhaes MA, Barbash IM, Ben-Dor I, Escarcega RO, Okubagzi PG, Baker NC, Chen F, Torguson R, Suddath WO, Staler LF, Pichard AD, Waksman R (2015). The impact of diabetes mellitus on outcome of patients undergoing transcatheter aortic valve replacement. IJC Metab Endocr..

[13] Conrotto F, D’Ascenzo F, Giordana F, Salizzoni S, Tamburino C, Tarantini G, Presbitero P, Barbanti M, Gasparetto V, Mennuni M, Napodano M, Rossi ML, La Torre M, Ferraro G, Omedè P, Scacciatella P, Marra WG, Colaci C, Biondi-Zoccai G, Moretti C, D’Amico M, Rinaldi M, Gaita F, Marra S (2014). Impact of diabetes mellitus on early and midterm outcomes after transcatheter aortic valve implantation (from a multicenter registry). Am J Cardiol.

[14] Tamburino C, Capodanno D, Ramondo A, Petronio AS, Ettori F, Santoro G, Klugmann S, Bedogni F, Maisano F, Marzocchi A, Poli A, Antoniucci D, Napodano M, De Carlo M, Fiorina C, Ussia GP (2011). Incidence and predictors of early and late mortality after transcatheter aortic valve implantation in 663 patients with severe aortic stenosis. Circulation.

[15] Puls M, Bleckmann A, Jacobshagen C, Danner BC, Hasenfuss G, Seipelt R, Schillinger W (2014). Diabetes increases short- and long-term mortality after transcatheter aortic valve implantation (TAVI). Dtsch Med Wochenschr.

[16] Elhmidi Y, Bleiziffer S, Piazza N, Hutter A, Opitz A, Hettich I, Kornek M, Ruge H, Brockmann G, Mazzitelli D, Lange R (2011). Incidence and predictors of acute kidney injury in patients undergoing transcatheter aortic valve implantation. Am Heart J.

[17] Nuis RJM, Van Mieghem NM, Tzikas A, Piazza N, Otten AM, Cheng J, van Domburg RT, Betjes M, Serruys PW, de Jaegere PPT (2011). Frequency, determinants, and prognostic effects of acute kidney injury and red blood cell transfusion in patients undergoing transcatheter aortic valve implantation. Catheter Cardiovasc Interv.

[18] Sinning J-M, Ghanem A, Steinhäuser H, Adenauer V, Hammerstingl C, Nickenig G, Werner N (2010). Renal function as predictor of mortality in patients after percutaneous transcatheter aortic valve implantation. JACC Cardiovasc Interv.

[19] Barbash IM, Ben-Dor I, Dvir D, Maluenda G, Xue Z, Torguson R, Satler LF, Pichard AD, Waksman R (2012). Incidence and predictors of acute kidney injury after transcatheter aortic valve replacement. Am Heart J.

