# Supplementary material for: Clinical impact of diabetes mellitus in patients undergoing transcatheter aortic valve replacement
Source: Cardiovasc Diabetol. 2015 Oct 1;14:131. doi: 10.1186/s12933-015-0291-3 (PMC4591728; doi:10.1186/s12933-015-0291-3)
Supplement: Supplementary file 1 — 10.1186/s12933-015-0291-3 Complication rates based on HbA1c levels. [file 12933_2015_291_MOESM1_ESM.docx]

Additional file 1: Table S1. Complication rates based on HbA1c levels

| **Complication** | **Diabetic (N=105)** | | **P-value** |
| --- | --- | --- | --- |
|  | **HbA1c<7%**  **(N=53)** | **HbA1c>=7%**  **(N=52)** |  |
| 30-days mortality | 0 (0) | 2 (3.5) | 0.25 |
| 6-months mortality | 1 (2) | 4 (7) | 0.19 |
| 1-year mortality | 2 (4) | 5 (9) | 0.23 |
| Cardiac tamponade | 2 (4) | 0 (0) | 0.23 |
| Valve malpositioning | 3 (6) | 2 (4) | 0.48 |
| Valve migration | 2 (4) | 1 (2) | 0.48 |
| Peri-procedural MI *(≤72 h post procedure)* | 0(0) | 1 (2) | 0.51 |
| Spontaneous MI *(≤72 h post procedure)* | 1 (2) | 0 (0) | 0.48 |
| Transient atrio-ventricular block | 2 (4) | 7 (13) | 0.08 |
| Permanent atrio-ventricular block | 4 (8) | 8 (15) | 0.19 |
| Permanent pacemaker implantation | 9 (18) | 8 (15) | 0.48 |
| New LBBB | 17 (33) | 9 (17) | 0.06 |
| New other conduction delay | 3 (6) | 5 (10) | 0.36 |
| New onset AF | 8 (15) | 6 (11) | 0.39 |
| AF episode in patient with history of AF | 2 (4) | 5 (10) | 0.23 |
| Heart failure post-procedure | 5 (10) | 7 (14) | 0.36 |
| AKI 3 | 1 (2) | 4 (7) | 0.18 |
| Total bleeding | 12 (23) | 10 (17) | 0.3 |

All values expressed as N (%)

MI – myocardial infarction; LBBB – left bundle branch block; RBBB – right bundle branch block, AF – atrial fibrillation; AKI- acute kidney injury.
